# Experimental Applications and Factors Involved in Validating Thermal Windows Using Infrared Thermography to Assess the Health and Thermostability of Laboratory Animals

**DOI:** 10.3390/ani11123448

**Published:** 2021-12-03

**Authors:** Antonio Verduzco-Mendoza, Antonio Bueno-Nava, Dehua Wang, Julio Martínez-Burnes, Adriana Olmos-Hernández, Alejandro Casas, Adriana Domínguez, Daniel Mota-Rojas

**Affiliations:** 1 PhD Program in Biological and Health Sciences [Doctorado en Ciencias Biológicas y de la Salud], Universidad Autónoma Metropolitana, Mexico City 04960, Mexico; cnrverduzco@hotmail.com; 2 División of Neurosciences, Instituto Nacional de Rehabilitación-Luis Guillermo Ibarra Ibarra, (INR-LGII), Mexico City 14389, Mexico; abueno@inr.gob.mx; 3 School of Life Sciences, Shandong University, Qingdao 266237, China; dehuawang@sdu.edu.cn; 4 Animal Health Group, Facultad de Medicina Veterinaria y Zootecnia, Universidad Autónoma de Tamaulipas, Victoria City 87000, Mexico; jmburnes@docentes.uat.edu.mx; 5 Division of Biotechnology—Bioterio and Experimental Surgery, Instituto Nacional de Rehabilitación-Luis Guillermo Ibarra Ibarra (INR-LGII), Mexico City 14389, Mexico; adrianaolmos05@yahoo.com.mx; 6 Neurophysiology, Behavior and Animal Welfare Assessment, DPAA, Xochimilco Campus, Universidad Autónoma Metropolitana (UAM), Mexico City 04960, Mexico; ale0164g@hotmail.com (A.C.); mvz.freena@gmail.com (A.D.)

**Keywords:** infrared thermography, laboratory animals, thermal windows

## Abstract

**Simple Summary:**

Laboratory animals are fundamental in biomedical science and monitoring their health and metabolic processes is essential to ensure their welfare and the quality of the research conducted. Infrared thermography (IRT) is a tool that can assess pathological or stressful states by measuring changes in the amount of heat that bodies dissipate. To date, some thermal windows have been utilized to evaluate animals’ thermoregulatory capacity, such as the orbital region, the auricle or ear pavilion region, the tail, and the interscapular zone where brown adipose tissue (BAT) is stored. However, the sensitivity and specificity of these windows are still subject to controversy because the scientific evidence that is available differs regarding the thermal information reported. Therefore, this paper aims to discuss the neurophysiological mechanisms of the vasomotor and thermogenesis responses (using BAT) in laboratory animals, the scientific usefulness of IRT, and the various thermal windows that are currently being used with laboratory animals.

**Abstract:**

Evaluating laboratory animals’ health and thermostability are fundamental components of all experimental designs. Alterations in either one of these parameters have been shown to trigger physiological changes that can compromise the welfare of the species and the replicability and robustness of the results obtained. Due to the nature and complexity of evaluating and managing the species involved in research protocols, non-invasive tools such as infrared thermography (IRT) have been adopted to quantify these parameters without altering them or inducing stress responses in the animals. IRT technology makes it possible to quantify changes in surface temperatures that are derived from alterations in blood flow that can result from inflammatory, stressful, or pathological processes; changes can be measured in diverse regions, called thermal windows, according to their specific characteristics. The principal body regions that were employed for this purpose in laboratory animals were the orbital zone (regio orbitalis), auricular pavilion (regio auricularis), tail (cauda), and the interscapular area (regio scapularis). However, depending on the species and certain external factors, the sensitivity and specificity of these windows are still subject to controversy due to contradictory results published in the available literature. For these reasons, the objectives of the present review are to discuss the neurophysiological mechanisms involved in vasomotor responses and thermogenesis via BAT in laboratory animals and to evaluate the scientific usefulness of IRT and the thermal windows that are currently used in research involving laboratory animals.

## 1. Introduction

Animal models are fundamental elements in scientific research that is conducted on numerous pathologies and in developing and testing of new drugs [1,2]. As most of the results that are obtained through the use of these animals are transposed to other species with similar physiologies, research protocols must include monitoring the animals’ physiological parameters in order to evaluate two key aspects: (1) their clinical health; and (2) the accuracy and veracity of the results that are obtained through the methodological procedures proposed [3,4].

Infrared thermography, or IRT, is one tool that has been adopted in research laboratories due to its ease of use and non-invasiveness [5]. IRT assesses the surface temperature of animals as it reflects changes in surface microcirculation [6]. Studies of various species have defined specific anatomical regions, called thermal windows, that allow researchers to observe the thermal changes and the amount of heat that is irradiated when the sympathetic nervous system (SNS) branch of the autonomic nervous system (ANS) is stimulated, and their effect on the core and surface temperature of the animals studied [7,8,9]. To date, IRT has been employed to determine the association between peripheral thermal alterations and negative states such as pain [6], emotions such as anxiety [10], and systemic circulatory changes derived from pathological processes, whether natural or induced. However, the information that these thermal windows provide varies significantly in the available literature so it is crucial to determine the differences that are observed among species, distinct environments, and individuals to ensure that interpretations of the thermograms that are captured are carried out as objectively as possible.

Therefore, this review aims to discuss the neurophysiological mechanisms of vasomotor responses and thermogenesis through BAT in laboratory animals as well as the scientific usefulness of IRT and the thermal windows of the tail, the ocular region, the auricular pavilion, and the interscapular region (BAT) in laboratory animals.

## 2. Importance of Thermoregulation in Laboratory Animals

Thermoregulation involves a series of physiological and behavioral mechanisms that operate to achieve thermoneutrality and maintain homeostasis [11], thus promoting an internal environment that is appropriate for the correct functioning of all an organism’s cells and organs [12]. In laboratory animals, it is vital to maintain normothermia with a constant, minimum metabolic rate because this promotes their welfare and, at the same time, ensures the quality and objectivity of the results that are obtained [13]. If an animal’s temperature is altered by the effects of thermal stress (cold or heat) then metabolic, cardiovascular, respiratory, and immunological alterations will follow. After all, the animals have little or no control over the temperatures they may be exposed to [14,15,16].

The factors that can directly affect the thermal biology of animals that are used in laboratories include characteristics of the microenvironment such as cage size and the materials they contain, the degree of transparency/opacity of the cage, bedding material, the number of animals per space (in groups or individually), acclimatization or quarantine periods, and levels of accumulated ammonia, among others [14]. Essential features of the macroenvironment are temperature, relative humidity, light/dark cycles, ventilation, and changes in airflow in the room and cage. Other factors to consider with maintaining the animals’ thermoneutral zone are handling procedures, confinement or constraint and the methods used, and any invasive or painful procedures to which they are subjected [17,18].

Vasogenic activity (vasodilatation, vasoconstriction), activation of brown adipose tissue (BAT), and shivering thermogenesis are the main neurophysiological mechanisms that are activated or inhibited in response to diverse thermal stimuli [19], and that provide information that is integrated into specific zones of the hypothalamus [9,20,21,22].

### 2.1. Peripheral Physiological Vascular Responses [Vasomotion of Cutaneous Blood Vessels]

Regulation of the peripheral circulatory system responds to the activation of the sympathetic nervous system (SNSi), which generates changes in the tone of surface cutaneous capillaries, veins, and arteries [23] to produce [warming] or dissipate heat [cooling] [24]. These changes proceed from the principal structure that is involved in thermoregulation; that is, the preoptic area of the hypothalamus (POA) [24], which contains thermosensitive neurons that react to changes in body temperature that are detected by peripheral receptors called transient receptor potential vanilloids (TRPV) due to external influences (e.g., extreme heat or cold) [25]. The ascending thermosensory pathways are activated once the thermoreceptors detect changes. This information is transmitted to the dorsal horn of the spinal cord and then to superior cerebral centers of the hypothalamus, including the lateral parabrachial nucleus (LPB) [26]. Depending on the stimulus type, neurons in the lateral subnucleus of the LPB are activated under cold temperatures, while neurons in the LPB dorsal subnucleus are activated by heat [25]. Once it enters these structures, the information is directed to the median preoptic nucleus (MnPO), which is in charge of exciting or inhibiting responses, depending on the nature of the stimulus (e.g., cold or heat). The MnPO modulates the conservation/production or loss of heat through diverse mechanisms that involve additional structures, such as the rostral magnus raphe, which transmits the signal to the sympathetic preganglionic efferent fibers of the intermediolateral columns of the spinal cord. These, in turn, innervate and provide vasomotor control of the cutaneous blood vessels and BAT [27,28]. Sympathetic cutaneous innervation generates two fundamental vasomotor responses for thermoregulation: vasodilatation and vasoconstriction [11], through cholinergic and noradrenergic innervation, respectively [29].

**Figure 1:**
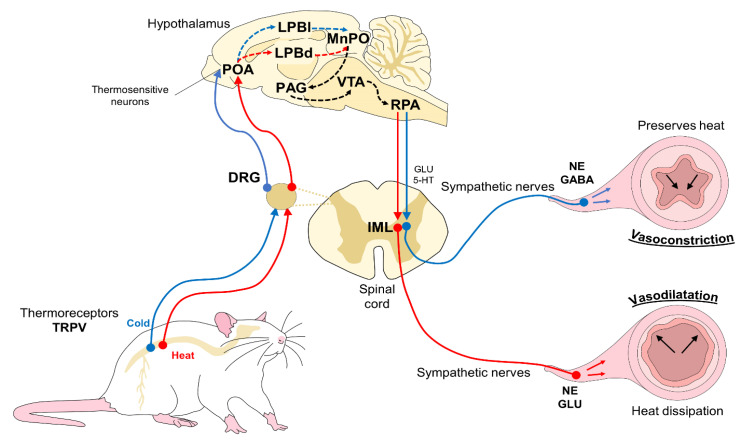
Vasomotor thermal response. Thermoreceptors in the skin of animals (mainly TRPV) detect hot or cold temperature changes and then send the thermal information to second-order neurons in the DRG. From these thermosensitive neurons, the stimulus is transmitted to superior cerebral centers, specifically the hypothalamus, and several areas inside it, such as the POA, LPBl (cool sensory neurons), and LPBd (warm sensory neurons), and other thermoregulatory centers, including the MnPO, PAG, VTA, and RPA. The latter is responsible for projecting the thermal signal from the brain to the IML of the spinal cord. Depending on the nature of the original stimulus and the influence of neurotransmitters such as NE, GABA, or GLU, the sympathetic fibers that innervate the blood vessels cause vasoconstriction to preserve heat when exposed to cold stress or vasodilation to dissipate heat and modulate heat stress. 5-HT: serotonin; DRG: dorsal root ganglion; IML: intermediolateral nucleus; GABA: gamma-aminobutyric acid; GLU: glutamate; LPBd: dorsal subnucleus of the lateral parabrachial nucleus; LPBl: lateral subnucleus of the lateral parabrachial nucleus; MnPO: median preoptic nucleus; NE: norepinephrine; PAG: periaqueductal gray; POA: pre-optic area; RPA: raphe pallidus; TRPV: transient receptor potential vanilloid; and VTA: ventral tegmental area.

Under hyperthermic conditions, or heat stress, vasodilatation—the reduction of sympathetic vasoconstrictor tone– and the consequent increase in blood flow are activated as a passive means of dissipating heat [28]. The above occurs in the so-called thermoregulator organs that promote the transfer of core body heat to the periphery or skin to prevent central overheating [27,30]. The characteristics of these organs allow them to efficaciously modulate thermal exchange because they tend to be zones with an ample corporal surface that are highly vascularized, have low or null hair density, and the predominance of arterio-venous anastomoses [11,31,32] (Figure 1).

Cases of these vasomotor responses can be observed in the thoracic and pelvic limbs and the tail of rats (*Rattus* sp.) and mice (*Mus* sp.) [14]. The blood vessels in the tail are of particular importance because the anastomosis between the main ventral artery and the lateral veins in the proximal portion of the tail facilitates heat loss [23,33]. The tail and extremities have also been seen to respond to stimuli from glutamatergic neurons (excitatory neurotransmitters that generate vasodilatation in the tail) [28]. In species such as rabbits (*Oryctolagus cuniculus*), meanwhile, which lack, or have very few, sweat glands, and the amount of insulator fur impedes efficient heat dissipation, vasodilatation of the arteries and veins near the surface of the ears constitute an alternative method of thermoregulation during events of heat stress [34]. Peripheral vasoconstriction, in contrast, is an autonomous mechanism that is activated when animals are exposed to cold stress [14]. When the organism detects decreases in body temperature, dermal microcirculation is reduced to conserve heat, lower the transfer of body heat to the environment, and redirect blood flow from the periphery to the central organs. To achieve the above is through the action of NE on the alpha-2 vascular adrenergic receptors, local inhibition of nitric oxide [9,29], and the action of GABAergic, an inhibitory neurotransmitter that produces vasoconstriction in the skin of the paw pads and extremities [28].

In rodents (rats, mice, etc.), vasoconstriction in the tail or the paws limits the amount of heat lost. However, even though both species utilize the same body regions, studies mention that differences exist between them such that the peripheral vasoconstriction response in rats is more efficacious due to their larger dimensions and greater body mass [14]. Research on rabbits has shown that exposure to cold reduces blood flow in the region of the ears (regio auricularis) (coefficient of variation from 62 ± 8% to 25 ± 4%) [32].

Under hypothermic conditions, vasoconstriction is the first mechanism that the SNA activates, but when this proves insufficient, the organism employs other methods of thermogenesis, although these entail increased energy expenditures [9]. They include heat production through shivering, non-shivering, and the effect of BAT [35].

Other physiological mechanisms (sweating, conduction, convection, radiation) also contribute to thermoregulation but depend on the species’ environment and anatomical characteristics [36]. For example, a mouse that weighs 30 g can balance its temperature through the vasoconstriction and/or vasodilatation of blood vessels in its tail. In contrast, in a 3.5 kg rabbit, these same processes may be insufficient and risk leading the animal towards a state that compromises its thermal equilibrium. In the case of laboratory animals, sweating is of little importance because of the scarce glandular tissue that is found in the region of the front and rear paws of mice [37], rats [38], and gerbils (*Meriones unguiculatus*) [39].

### 2.2. A Non-Shivering Mechanism of Thermogenesis: BAT

**Figure 2:**
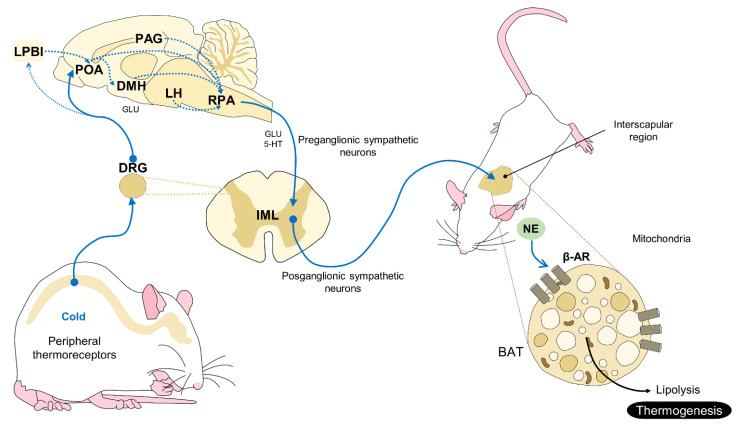
Non-shivering thermogenesis by BAT. This response begins with the activation of peripheral thermoreceptors that detect a decrease in surface temperature. The thermal signal from these receptors reaches the DRG of the spinal cord and is further processed in hypothalamic brain areas such as the LPBl, which projects neurons towards the POA and the other structures that are involved in thermoregulation (PAG, DMH, LH, RPA). GLU, an excitatory neurotransmitter that promotes sympathetic action on BAT via sympathetic preganglionic fibers from the RPA, is also involved in this process. Once in the IML of the spinal cord, the postganglionic sympathetic fibers that innervate the adipose tissue interact with the NE that is released by the SNSi, and then bind to the β-AR of the BAR cells to produce lipolysis and generate caloric energy. 5-HT: serotonin; DMH: dorsomedial hypothalamus; DRG: dorsal root ganglion; IML: intermediolateral nucleus; GLU: glutamate; LH: lateral hypothalamus; LPBl: lateral subnucleus of the lateral parabrachial nucleus; NE: norepinephrine; PAG: periaqueductal gray; POA: pre-optic area; RPA: raphe pallidus; and β-AR: beta-adrenoreceptors.

Brown adipose tissue (BAT) consists of fat deposits in specific body regions of small mammals that play a fundamental role in thermogenesis. BAT in rodents is found in the interscapular, subscapular, axillary, peri-renal, and periaortic regions [40]. BAT is considered the principal mechanism of non-shivering thermogenesis in these animals, because its metabolic activity produces heat and is controlled from the hypothalamus by sympathetic action through noradrenergic fibers [41] and the presence of specialized proteins that give this brown fat its heat-generating capacity [42]. The regions of the POA that are involved in the vascular response that is described above also participate in activating BAT, as do neurons in the median preoptic area (MnPO) of the medial preoptic (MPO) and lateral preoptic (LPO) areas; specifically, the dorsomedial (DMH), paraventricular (PVH), and the ventromedial (VMH) hypothalamus [42] (Figure 2).

When species, such as rodents, are exposed to cold temperatures, thermosensitive neurons in the hypothalamus trigger the sympathetic release of norepinephrine (NE). BAT contains β-adrenergic receptors that are activated by NE, resulting in the rupture of triglycerides in the BAT and the lipolysis of free-fatty acids. These acids interact with cytosolic proteins that stimulate the mitochondria in the BAT to produce energy and, with that, heat [43].

Thermogenesis by BAT is an essential mechanism in small newborn mammals and rodents, for maintaining core temperature (Tcore) through the catabolism of brown fat [14]. In extreme cold situations, this constitutes a thermoregulatory response of greater efficacy than thermogenesis via shivering because the limited muscle mass of those animals does not permit sufficiently intense shivering to maintain thermal stability. If prolonged, this is a condition that can prove fatal [14,19]. In addition, behavioral adaptations precede various physiological responses that contribute to normothermia [44]. These processes reduce energy expenditures by activating the autonomous mechanisms that were mentioned previously [45]. This behavioral modulation is the first phenomenon that is observed. Some examples are when animals attempt to conserve heat by building nests, adopting postures such as huddling or grouping with congeners [44,46], or remaining immobile in cold environments [46,47]. In contrast, under conditions of excess body heat, animals tend to increase their activity or seek to augment heat exchange by spreading saliva on their fur and ingesting larger quantities of water [32].

To a certain degree, these behavioral adaptations function to maintain animals in a zone of thermoneutrality. However, the autonomous and vascular changes that are described above are activated when the temperature threshold reaches a certain point, and these measures are insufficient to ensure a return to thermoneutrality [9].

## 3. Thermal Windows Used with Laboratory Animals

Those vascular changes, and the activation of BAT with the consequent heat production in the interscapular region, are events in which infrared thermography (IRT) has emerged as a valuable tool for assessing animal welfare [48]. IRT can detect temperature modifications in response to events that generate stress or pain or due to environmental influences [33,49,50]. Specific anatomical areas of the body, denominated thermal windows, have been identified with characteristics such as a high density of surface blood capillaries, arteriovenous anastomosis, and the absence of hair, which facilitates temperature loss or gain by changing the diameter of the blood vessels [51,52]. In laboratory animals, the body regions that comply with these conditions include the tail, ears, eyes, interscapular region with BAT, and the paws, as shown in Table 1. However, discussions are ongoing about the usefulness and validity of these regions in all the species that are utilized in research [10,53]. The following sections describe each of these thermal windows in detail and interpret the information that was obtained from each one concerning various recent experimental models.

### 3.1. Thermal Windows: The Tail

**Figure 3:**
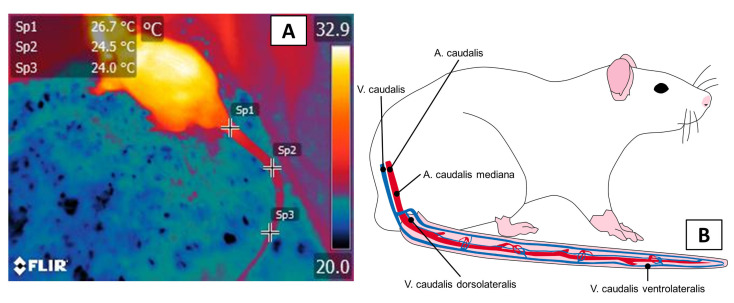
The thermal window of the tail in rodents. (**A**) this window consists of three spots that are located in the proximal, middle, and distal parts of the tail. Temperature average values can be obtained for each one of these focal points. (**B**) vascularization in the caudal region. In this zone, vasculature is provided mainly by the branches of the caudal aorta artery, such as the arteria caudalis mediana, the vena caudalis dorsolateralis, and the caudalis ventrolateralis. A: artery; V: vein.

In rodents, the tail (regio caudalis, cauda) is one of the main thermal windows that is involved in thermoregulation because it features vascularization by large blood vessels that come from the coccygeal artery (arteria caudalis (coccígea) mediana), which presents a projection into the ventral region of the caudal vertebrae, as Figure 3 shows [54]. These vascular characteristics permit an easy dissipation of up to 25% of body heat in climates with extreme heat [14,55,56]. This window is of great interest because most rodents use the tail as an extension that helps them maintain thermostability [11].

In rodents, applying IRT to the tail has emerged as a technique that allows researchers to determine core temperatures, as Fiebing et al. [57] showed in their study. Those authors compared IRT to invasive methods for evaluating temperature (intraperitoneal, rectal, subcutaneous) in 430 nude NMRI mice. The rectal temperature readings and IRT in the tail showed similar mean values (38.05 ± 0.46 °C), while the intraperitoneal data loggers showed low mean values (36.57 ± 0.59 °C), and the subcutaneous transponder recorded values 2.21 °C higher. The rationale for using this window is that exposure to adverse conditions, such as extreme cold, the immediate vasomotor response takes the form of peripheral vasoconstriction, intending to reduce heat loss into the environment [14,58].

In contrast, IRT in pigs (*Sus scrofa*) has shown a tendency to underestimate the minimum rectal temperature values with a proportional bias of 0.8 °F—(+/−) [59], in contrast to what has been observed in other species, such as Rhesus monkeys (*Macaca mulatta*) [60]. Despite these differences among species and underestimates of real body temperature values, the tail temperature is suggested as an option for determining Tcore because this region provides information that is associated with autonomous nervous system (ANS) activity [6].

In social stress or fear situations that are induced by electrical discharges, Vianna and Carrive [61] observed that the temperature of the tail and paws decreased significantly (−5.3 and −7.5 °C, respectively), contrary to observations of ocular regions, the head, and the back, where increases of 0.8–1.5 °C were recorded. This response was attributed to sympathetic vasoconstriction that reduced peripheral blood flow. Bitar et al. [33] reported a similar phenomenon in their evaluation of 87 adult Sprague-Dawley rats in which they measured the vasomotor response that was associated with pain that was induced by nociceptive thermal stimuli when the animals were awake but anesthetized with 2.5% halothane. When those animals perceived the harmful stimulus, the heart rate (HR), mean arterial pressure (MAP), and Tcore all increased significantly (45 beats per minute, 46 mmHg, and 0.7 °C, respectively, *p* < 0.05), while the temperature of the tail decreased by 0.7 °C due to the vasomotor thermal response to the nociceptive stimulus applied.

**Figure 4:**
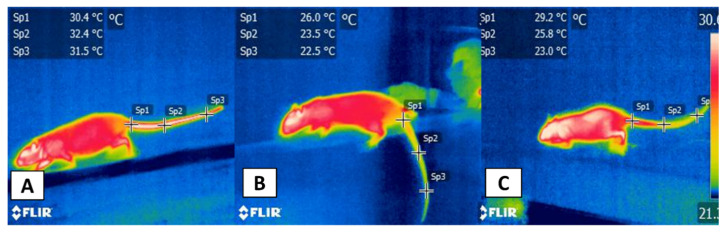
Thermal response in a rat’s tail in an experimental model of a cerebrovascular event during the beam walk test. (**A**) before surgery: a 300-g male Wistar rat on the 7th day of training. The average temperatures of the proximal (Sp1), medial (Sp2), and distal (Sp3) regions of the tail were 30.4, 32.4, and 31.5 °C, respectively, before induction of the brain injury with iron chloride. (**B**) Rat at one-day post-stereotaxic surgery. The left hind limb slips due to the cerebral motor injury. The three average temperatures of the tail show reductions of 4.4, 8.9, and 9 °C, respectively. (**C**) Rat at seven days post-surgery, one week after the cerebrovascular event. The tail maintained low mean values (1.2, 6.6, and 8.5 °C, respectively) compared to the baseline values in image (**A**). This vasoconstrictor response represents the activation of the SNS and the catecholamines action due to the pain perception that resulted from the brain injury.

This phenomenon can be explained by the neurosecretion of catecholamines, such as NE and epinephrine, which causes peripheral vasoconstriction in zones such as the tail of rodents [62,63], as the authors observed in preliminary studies that were conducted with male Wistar rats using a model of the cerebrovascular lesion, as shown in Figure 4. In this regard, Gordon et al. [55] reported that the thermal response of the tail increased in rats during the open field stress test and after induction of fever by administering lipopolysaccharides (LPS) due to the activity of the SNSi.

Similarly, a study of 16 male Wistar rats that analyzed the thermal response of the tail associated with stress that was induced by handling. Those authors recorded temperatures in this region before, during, and after handling. They found that the temperature that was recorded was significantly lower in the animals during handling compared to that of the control group (27.7 vs. 26 °C, *p* < 0.001) [64]. The preceding demonstrated the autonomous vasoconstrictor effect was triggered by stressful events. In contrast to that report, however, a study of 18 naive Wistar rats that evaluated the response of HR, MAP, and IRT in the tail after microinjections of orexin-A in the medullar raphe, observed that the administration of that substance caused a significant increase in HR (+99 beats per minute) and MAP (+11 mmHg), but did not significantly change the temperature of the tail [65]. Likewise, Lecorps et al. [66] measured the thermal response in the tail and body surface of mice that were exposed to the odor of predators that contained the component 2,5-dihydro-2,4,5-trimethylthiazole (TMT). In that study, the mice responded atypically with a discrete increase of body and tail temperatures recorded from 2 min after initiating contact with the stimulus.

The discrepancies that were observed in the studies that have used the tail as a region for evaluating IRT have been suggested be that, due to its vascularization, the thermal window of the tail is a site that responds quickly to local vasomotor changes derived from sympathetic activation during potentially stressful events such as harmful stimuli or thermal stress. However, additional studies are required to corroborate the usefulness of this zone for the non-invasive measuring of Tcore since its sensitivity to environmental changes can generate underestimates of real values.

### 3.2. The Ocular Window

**Figure 5:**
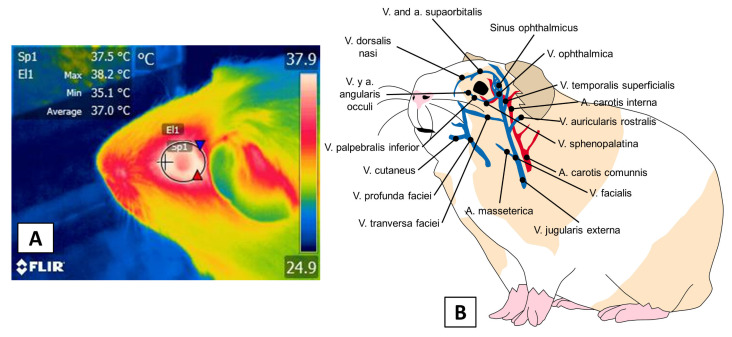
Orbital thermal window. (**A**) this window is indicated in the software by a circle approximately 3 cm in diameter. The shape must include the ocular globe and eyelids. (**B**) anatomical composition. The ocular blood supply is carried through the v. and a. supraorbitalis, and the v. and a. angularis occuli, two branches of the v. ophthalmica.

The ocular region [regio orbitalis] is another window that has been studied in laboratory animals, such as rabbits, guinea pigs, rats, and mice. Figure 5 illustrates the vascularization of the eye, especially two large arteries—the arteria supraorbitalis and angularis occuli—that are ramifications of the facial artery which supplies circulation to this region. One aspect that must be emphasized is that the arteria angularis occuli is innervated by the facial nerve [54], so that zone responds sensitively to the predominant autonomous tone during diverse stimuli that is applied to species such as large ruminants [51], dogs (*Canis lupus familiaris*) [67], and equines [68].

Gjendal et al. [69] evaluated the eye and tail temperatures during three potentially stressful events: a maze test, an intraperitoneal injection, and under anesthesia with isoflurane for one minute in 80 males C57BL/6 rats. They found that the test that generated the most evident stress response was anesthesia with isoflurane, as the IRT registered reductions (as significant as 3.5 ± 0.5 °C) in the tail, eye, and body temperatures. In that case, the ocular temperatures remained 1.5 °C lower than those of the tail. Meanwhile, Vogel et al. [53] compared rectal and ocular temperatures in rabbits, rats, and mice, determining that temperature measurements of the ocular surface of the rats were significantly higher than rectal readings at 36.5 ± 0.2 °C and 35.7 ± 0.1 °C, respectively. These results are similar to those that were obtained in rabbits (39.1 ± 0.2 °C ocular, 38.2 ± 0.2 °C rectal). The authors concluded that temperature readings at the ocular level could be a reliable thermal window for comparisons with body temperature since they did not observe modifications in the readings before events that generate stress (such as constraint). Similarly, a study of 20 guinea pigs of both sexes, but different ages, used ocular IRT to quantify surface thermal changes in animals that were subjected to interaction tests with and without conspecifics and with humans. Their data showed that the temperature of the eye increased by 0.36 ± 0.40 °C (*p* < 0.001) during interaction with humans, contrary to responses that were observed in other species [70].

In other work, Stewart et al.’s [71] descriptions of bovines under nociceptive stimuli indicate that temperature at the ocular level presented an initial increase followed by a decrease in response to the sympathetic secretion of catecholamines that caused peripheral vasoconstriction. In this regard, a study of 16 C3H mice that was based on a 30-min cage test evaluated behavior and orbital temperature. As the mice presented behaviors that were associated with stress (e.g., reduced activity, anorexia, or group improvement), the ocular IRT readings decreased, maintaining a negative correlation (Spearman *p* = 0.34, *p* = 0.0001) [72].

**Figure 6:**
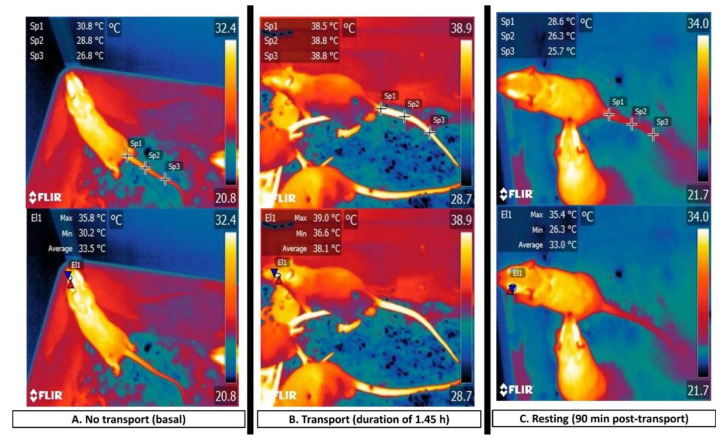
Comparison of the influence of transport on thermal responses in the ocular and tail regions of laboratory rats. In this trial, three phases were designated: (**A**) non-transport (basal); (**B**) transport for 1.45 h. The average ambient temperature inside the vehicle was 33 °C, and the average surface temperature of the plastic boxes (50 × 40 cm) was 31 °C; and (**C**) resting (90 min post-transport). The comparison of the differences among temperature readings according to the three study phases revealed that the average recordings for the tail (Sp1) and ocular surface (El1) during phase (**B**)–transport– increased by 7.7 and 4.6 °C, respectively, compared to basal values (30.8, 33.5 °C). In contrast, during the resting phase (**C**), the tail temperature decreased by 2.2 °C, while the average ocular temperature returned to its basal level of 33 °C with a difference of only 0.5 °C. In another moment of the analysis, the thermal windows showed the average temperatures (Sp1) of 30.8 and 33.5 °C, respectively, in the caudal and ocular regions (El1) in the non-transport phase. These values increased to 38.5 and 38.1 °C, respectively, during phase (**B**). In phase (**C**), significant differences were observed in the average values for both the tail and eye with readings of 28.6 and 33.0 °C, respectively. Eye temperatures returned to values that were similar to those that were reported at rest. This preliminary study indicates the importance of selecting thermal windows following the sensitivity of each region.

Findings of this kind allow us to establish that, when compared to the tail window, the ocular window presents greater vascular sensitivity to the circulatory changes that result from diverse stimuli that are considered negative for animals, as in the case of psychosocial stress [62] or during the perception of pain [6]. This effect is illustrated in Figure 6, which presents preliminary study results that were conducted by the authors that compared the thermal response of the ocular surface and the tail before, during, and after laboratory rats were transported to a bioterium.

The scientific evidence that has been reported to date shows that, thanks to its rich vascularization and autonomous innervation, the ocular window is sensitive to vasomotor changes that are associated with stressful events, in contrast to the tail. Nonetheless, this window also has limitations; for example, the amount of tearing and the degree of evaporation are negatively associated with the increase in temperature [73]. However, the vascular response also depends on the degree and intensity of the stressful stimulus (e.g., response to fear or anxiety) [74], so these factors must be considered when attempting to validate this region.

### 3.3. The Auricular Pavilion

**Figure 7:**
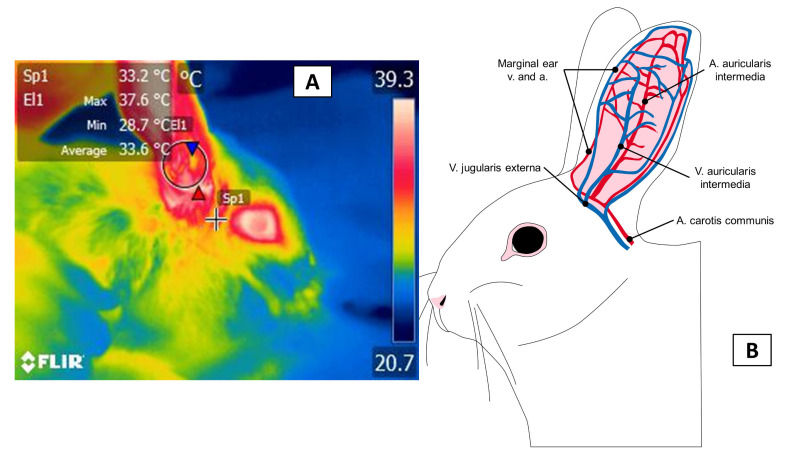
Auricular thermal window. (**A**) a circle of approximately 4 cm covers the central region of the pinna to register the temperature coming from the inner ear and tympanic membrane; (**B**) vascularization of the regio auricularis. The irrigation of this region is provided by the arteria auricularis intermedia and the marginal arteries and veins from the vena jugularis externa.

The auricular region is another zone where numerous blood vessels permit observing circulatory changes in diverse experimental designs and laboratory species. The external jugular vein and the external carotid artery supply irrigation to this zone [75]. The central ear artery and the marginal ear veins are the principal vessels that are involved in thermal control in this zone [76] (Figure 7). In the presence of diverse stimuli, the vasculature of the ear responds to the sympathetic influence that controls vasomotor tone in arteries, arterioles, venules, and arteriovenous anastomoses that dissipate larger or smaller amounts of heat depending on the blood flow [77]. These changes in the amount of heat that an animal emits can be observed by IRT in the pinna, where they appear as temperature increases when vasodilatation occurs or decreases due to the effect of peripheral vasoconstriction. These responses facilitate the process of thermal evaluation in animals [78].

In the case of rabbits, the size and dense vasculature of their ears have led to laboratory studies of the circulatory changes in that region that are produced by various procedures and stimuli using IRT. A pilot project with six hybrid rabbits analyzed the effect of emotional stress on the internal temperature of the auricular pavilion, the ocular, and the periocular skin for five days [77]. The animals were exposed to various social stressors, including coexistence with congeners in a new environment, loud noise, and three minutes of immobilization. In response to all of these stimuli, especially during immobilization, auricular temperatures decreased by more than 2 °C. At the same time, a comparison with cortisol levels showed a proportional relationship between the temperature increase that was detected by IRT and the concentration of this biomarker (from 47.80 ng/mL basal to 80.16 ng/mL) [77].

**Figure 8:**
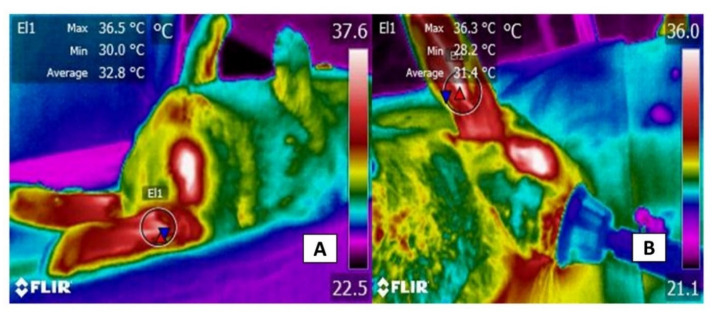
Comparison of the thermal response in the auricular region of rabbits that were subjected to two methods of anesthesia. (**A**) injectable anesthesia with xylazine and ketamine. The pinna shows maximum, average, and minimum of 36.5, 32.8, and 30.0 °C, respectively. (**B**) inhaled anesthesia with isoflurane. The thermal response of the ear registered maximum, average, and minimum temperatures of 36.3, 31.4, and 28.2 °C, respectively. From a comparative perspective, the inhaled anesthetics generated greater peripheral vasodilation that promoted more heat loss than the injected anesthesia, with a difference of 1.8 °C in body regions such as the rabbits’ ears. This vasodilator response can lead to perioperative hypothermia, an effect that can be detected early using IRT.

Hutu et al. [79] also used IRT in the ear scapha, between the apex and tragus, in 14 white New Zealand rabbits to compare those measurements to rectal temperature values after orthopedic surgery on the meniscus and cartilage to simulate trauma. The study found that the temperature of the ear correlated positively with body temperature (r = +0.579), as the latter averaged 39.03 ± 0.7 °C, while the former averaged 37.50 ± 0.12 °C. Similarly, hypothermic processes were detected in 10 rabbits in both body temperature and IRT (37.93 and 35.73, respectively). These findings led the authors to conclude that IRT can be used with this thermal window to estimate body temperature objectively. In this regard, the evaluation of the body temperature is a key parameter to evaluate and determine the health status of animals. The gold standard for its measure is through rectal temperature; however, this method is considered stressful due to the handling. Hence, IRT is a complementary method to correlate body surface temperature to core or body temperature in a non-invasive way [80]. Another study with rabbits determined the effect of room temperature (cold or hot) on the thermoregulation of leporids. There, 130 rabbits were housed in two rooms with different temperatures (20–30 °C in room A, above 31 °C in room B to simulate heat stress). The authors monitored the external and internal ear temperatures and the ocular and nasal regions for one year. Although it was possible to observe the increase in circulation due to heat stress and cortisol concentrations for both windows, only the ocular window showed significant differences (up to 3.36 °C). However, the internal temperature of the ear was more suitable to measure the thermal response due to environmental influence. Although the circulatory effect in room B was consistent with heat stress and increased cortisol concentrations, they reported that only the ocular window showed significant differences (up to 3.36 °C), but that for measuring the thermal response to environmental influences, the internal ear temperature was the most suitable. However, they suggested that the minimal ocular and nasal temperatures are better windows [81]. This relationship between temperature increases in certain body areas, such as the ocular window and core temperature, and the reduction in the amount of heat that is irradiated in the auricular window has been demonstrated in preliminary results of a study carried out by the authors illustrated in Figure 8. The study of New Zealand rabbits used this window to evaluate the impact of anesthetics on the temperature in this region. For example, in other studies, Luzi et al. [82], after rabbits were subjected to an immobilization procedure for 15 min, the internal ear pavilion temperatures decreased from 35.3 to 34.8 °C, while the ocular and rectal temperatures registered the opposite effect. Finally, measurements of the pinna of rabbits a few days after birth were lower due to vasoconstriction to conserve heat. At the same time, temperatures of the interscapular region and BAT increased, reflecting the limited thermoregulation capacity that is characteristic of newborns [83].

IRT has been employed in rodents, such as mice, to evaluate routine intraperitoneal (IP) drug administration techniques. In 114 mice, ear temperatures increased significantly after applying four distinct methods of constraint to administer a saline solution via IP [84]. The four methods of subjection were: a control group where the tail held the mice; a group where they were held with the head hanging down; a third where the head was held and inclined upwards; and a fourth where the Baek method was used with the animal holding onto a surface with its front paws while the researcher raised its rump and tail to locate the appropriate abdominal quadrant. All four techniques raised corticosterone levels, especially method three (head inclined upwards, approximately 1500 mmol/L). Although temperature increases were observed in the ocular region and decreases were recorded in the tail (indicative of stress responses), it was impossible to establish a relationship between the ears and the procedure or corticosterone concentrations [84]. The authors concluded that the inconsistencies that were registered were a species-specific factor that can alter the sensitivity and specificity of IRT.

Rabbits and guinea pigs present arteriovenous anastomosis in the ears, while rats and mice lack this feature. Instead, they use capillaries that permit thermal exchange, though in a way that is less efficient than in rabbits. Hence, it has been suggested that the auricular pavilion window of rats and mice should not be utilized to evaluate acute stress. A similar case of IRT use in mice has been observed in research by Xu et al. [85], who measured changes in segmental warming in several body regions (ears, paws, and tail) of 10 C57BL/6 mice that were subjected to epidural anesthesia with bupivacaine. The animals that received bupivacaine, compared to the control group with a physiological saline solution, the temperatures of the pelvic limbs increased by 3–4 °C (with a mean of +3.73 ± 1.56 vs. +0.56 ± 0.68 °C). The authors also took temperature readings of the ears and thoracic limbs; however, the changes in those zones were minimal and not significant, suggesting that only in the hind limbs was it possible to observe the selective vasodilator effect due to the loss of sympathetic vasomotor control produced by the epidural technique.

Larger species, such as ruminants or pigs, are other animals that are used in diverse experimental protocols, including some that have utilized IRT to detect hyperthermia or hypothermia. Examples of this are viral infection models with the type 2 bovine diarrhea virus in 15 Angus-Hereford heifers (*Bos taurus*). In those animals, IRT measured the mid-ventral area of the ear but did not show any differences until five to six days post-inoculation, in contrast to the ocular temperature. Although the auricular window did not register an increase in the first few days, by day 10, temperature increases were observed: from 22.38 °C (basal) to 26.28 °C. This indicates that IRT is a sensitive tool for detecting hyperthermic states for the auricular region, but that has low specificity because of the time that is required to detect significant differences [86]. Likewise, experimental studies with 80 piglets aged 28 days and fed diets enriched with plant additives (*Passiflora incarnata*) found temperature increases in the ear and on the back (*p* < 0.01), compared to piglets that received traditional diets. These values, together with improved immune function, growth performance, and lower incidences of lesions (*p* < 0.01), are parameters that, if employed at the same time as IRT, make it possible to evaluate the welfare of animals such as piglets [87]. Also, in other mammalian newborns whose capacity for thermoregulation is limited and must be controlled in a laboratory environment to prevent alterations in the results to be analyzed.

### 3.4. The Interscapular, or BAT, Window

In small mammals, newborns, or species that hibernate, the first mechanism that is activated under extremely cold conditions is non-shivering heat production that is generated by the activation of BAT [83]. In these animals, 40% of BAT reserves are located in the interscapular region and zones around the neck, reflecting the inadequate thermoregulatory capacity of altricial mammals, though the amount of BAT decreases [10,83].

**Figure 9:**
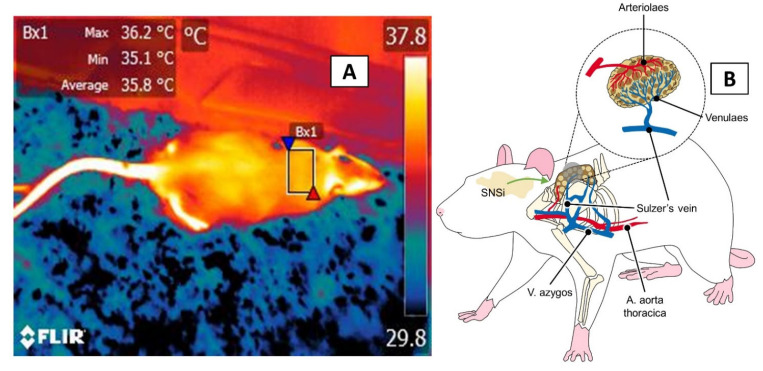
The interscapular, or BAT, thermal window. (**A**) A rectangle of approximately 3 cm is drawn in the dorsal area over the interscapular space to measure the temperature in this region (**B**) anatomical consideration. This window has dense vasculature, but its main blood vessel is the Sulzer’s vein, which supplies the brown adipose tissue. Under cold temperature conditions, rodents activate non-shivering thermogenesis to increase their body temperature and the heat irradiated from the BAT region.

Thermoregulation via BAT responds to the sympathetic activation of the β-adrenoreceptors that are located in the brown adipocytes and to a dense vasculature that provides oxygen, removes CO_2_ and products of cell metabolism, and, primarily, participates in thermogenesis through the Sulzer vein, which connects to the heart and other venous return towards the vertebral sinus [88]. The influence of the SNSi on adrenergic receptors in the BAT promotes lipolysis of free fatty acids and, with that, the production of heat [89] (Figure 9). The role of sympathetic tone and the effect of NE on BAT has been demonstrated in studies that induced BAT’s caloric activity by injecting NE or have inhibited its activity by administering receptor antagonists (e.g., propranolol) [90]. Its activation, however, requires consuming oxygen, which can impact animals’ growth and survival [10].

As mentioned previously, one of the properties of BAT is to generate heat during periods of extreme cold and thermosensitive stages of mammals or neonates. Research in this area has been conducted with rabbits to determine BAT’s role in the thermoregulation of newborns. In 12 rabbit pups of the Hyplus strain (New Zealand×Californian), IRT was used in the interscapular zone to determine BAT’s role in thermoregulation under cold conditions. In that study, Gilbert et al. [83] evaluated the thermal responses of non-insulated 3–4-day-old rabbit pups and insulated 10–11-day-old rabbit pups, that were generated by exposing them to environmental temperatures of 23 °C for two hours, and 14 °C for one hour, to analyze the importance of huddling as a means of maintaining heat. The authors found that the temperature of the interscapular zone in cold conditions was higher in the pups in the group that huddled than in the pups that were housed alone (35.6 ± 0.4 vs. 30.9 ± 0.9 °C). They further observed that the BAT, ear, and body temperature maintained a correlation in the 3–4-day-old pups (R^2^ ≥ 0.95, *p* ≤ 0.025). At the level of physiometabolic functioning, this entailed a more significant energy expenditure by the newborns due to their inadequate thermoregulatory capacity [83]. Another species that has limited thermoregulation in the first days of life is the pig. While the role of BAT in piglets has not been evaluated, Boileau et al. [91] observed that the dorsal region posterior to the neck presented temperature decreases in 46 pigs that was associated with agonistic behaviors, such as fights between congeners.

A similar case involving 24 male Wistar rats showed that although IRT that was applied in the interscapular window is not an adequate tool for evaluating states of stress that are induced by conditioned fear, it is a good indicator of the thermal stress that animals may suffer. In the study by Marks et al. [10], exposure to cold (4 ± 2 °C) for 30 min generated gradual increases of BAT up to a maximum of 34.14 ± 0.17 °C, together with a stabilized increase on the back temperature and reduced infrared temperature reading of the tail of up to 8.69 ± 0.49 °C. That study also reported that administering propranolol impaired the thermoregulatory capacity of BAT under cold conditions and reduced the amount of heat that was generated and dissipated by that zone (by +0.7 °C). Also, in rats, administering an agonist of the β-receptors of BAT increased IRT readings in the BAT region that were related to the thermograms that were recorded in the tail, eye, and inner ear. This led to the observation of an increase in the heat amount that dissipated in the tail (vasodilatation), that did not affect the ear or eye temperature due to the effect of BAT thermogenesis and the increase in body temperature [89].

IRT has also been applied in surgical procedures of the spinal column in mice to non-invasively evaluate the efficacy of one-drug analgesic protocols (buprenorphine) versus multimodal analgesia with carprofen and buprenorphine in controlling acute post-operatory pain. Radaelli et al. [92] evaluated IRT in the interscapular region and tail using the Mouse Grimace Scale (MGS) scores to measure pain. Thermographs were taken only of the BAT region because of the large variations in tail temperatures. In the BAT, the patients who received two drugs showed lower temperatures than those that were given only one analgesic (36.5 vs. 39.9 °C). Those recordings concurred with the mean scores on the MGS for the facial action unit of the ears (1.5 points). The study showed that the IRT functions not only to determine the thermal stability of animals and the influence of the sympathetic activity in response to a stressor but can also contribute to refining surgical techniques and pharmacological protocols to reduce pain and improve the welfare of laboratory animals that are subjected continuously to various experimental procedures.

## 4. Areas of Opportunity and Future Directions

**Figure 10:**
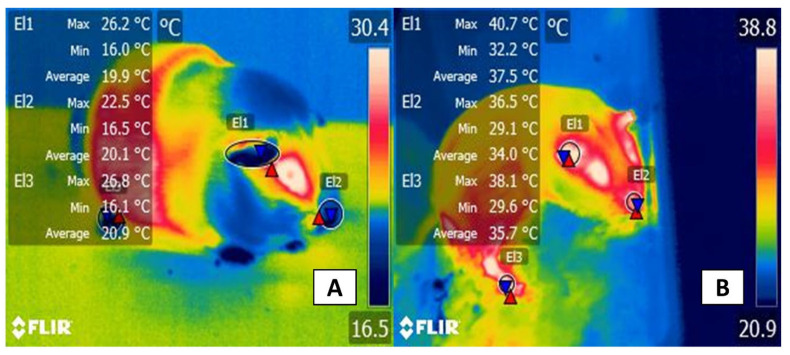
Guinea pigs were exposed to two conditions of thermal stress. (**A**) an adult male Dunkin-Hartley albino strain guinea pig (750 g) with hypothermia. The animal was transferred for identification from its housing box (with a controlled environment) to an operating room with no heating system. The mean temperature of the nasal region (El2) is 20.1 °C; of the ear (El1), 19.9 °C; and of the tarsi of the right pelvic limb (El3), 20.9 °C. (**B**) a guinea pig with hyperthermia during the post-surgical recovery period with heaters. The temperature recorded around the box was 40 °C. After 30 min of exposure to the heat source, mean temperature increases were observed in the nasal (El1) and auricular (El2) and in the tarsal region of the pelvic limb (El3): 37.5, 34.0, and 35.7 °C, respectively. This represents the impact that extreme environmental conditions can produce on the thermoregulatory capacity of animals during routine procedures and the consequent activation of the hypothalamic centers to control body temperature.

As with other species, in laboratory animals, the use of IRT requires considering and controlling certain factors that can directly alter thermographic recordings. Researchers who work with laboratory animals constantly seek to refine their techniques by adopting non-invasive technologies, such as IRT, and minimizing variation in the parameters that are evaluated to report results as significant and representative as possible. Reducing the environmental variations that can influence IRT readings requires strict control of such factors as room temperature, exposure to direct sunlight, activity levels of the animals involved, the amounts and kinds of food ingested, and circadian cycles (e.g., taking temperatures at the same hour every day during experiments) [86]. Most studies of rodents, for example, maintain the rooms at constant temperatures of 22–24 °C to reduce the effect of environmental temperature and humidity on IRT in all thermal windows [10]. Figure 10 exemplifies the influence of the environment (hypothermia, hyperthermia) in an experimental model with guinea pigs that was based on preliminary studies by the authors.

With regards to these thermal windows, it is essential to work with a body region that provides researchers with specific information on the activity of the SNSi, its relation to stressful, inflammatory, pathological, or nociceptive processes, and the microcirculatory response that results from the perception of social, thermal, or physical stimuli [33,71,93]. In addition to the windows that were discussed above, other body regions at the thoracic level have been used with IRT to non-invasively generate information on systemic cardiac and circulatory activity [94]. This use of IRT provides the opportunity to include this tool as an additional technique for the peri-operatory monitoring of physiological parameters that are based on slight changes in surface temperatures [63].

In mice and rodents more generally, IRT has begun to be utilized as a method for refining experimental designs and determining the humane endpoint of the lives of laboratory animals based on body temperature. The above is important because it eliminates the need for invasive procedures such as taking rectal temperatures or implanting subcutaneous thermal transponders. One such case involved an experimental model of acute illness and fever due to endotoxemia, where subcutaneous temperatures and IRT images of the anogenital region showed correlations, suggesting that the latter is a viable, non-contact option for predicting the mortality of laboratory animals [95]. In addition, IRT has been employed to study alterations in hypervascularity and angiogenesis around tumors, even some that had not been detected clinically, since it was able to detect tumors based on temperature changes of 0.1 °C in the stages of tumor development [96].

Recently, Vainer [97] proposed that sorption-enhanced IRT can be a method for the remote monitoring of the respiratory function in rats, pigs, and humans by evaluating respirations and the intervals between them with a sensitivity 4.5–250 times greater than conventional infrared monitoring systems permit.

Finally, to consider IRT a valid instrument for evaluating animal welfare and thermal indices [98], we must consider the species [99] and the resolution that is necessary to capture the phenomena in animals as small as mice, for which a resolution of 640 × 480 is recommended. In addition, standardizing procedures requires machines with autofocus and fixed cameras instead of handheld models [57] and automatic methods for analyzing thermograms and improving computer-generated segmentation for clinical interpretations and the application of those analyses [100]. Today, thermographic equipment is available at affordable prices, such as the cost of a smartphone, with integrated lenses such as FLIR One, which allow their use in diverse laboratories to evaluate temperature changes in mammals or ectotherms such as lizards [101]. The literature that is available on this technology is, however, still limited. It is necessary, in any case, to evaluate the degree of sensitivity and specificity of these tools for their possible validation and practical implementation due to confounding factors such as perceptions of negative emotions that can alter temperatures [72]. Table 1 summarizes the current findings regarding the use of IRT in some of the studies that were included in this article involving laboratory species and different thermal windows to evaluate thermoregulation and animals’ reaction to a particular stimulus.

## 5. Conclusions

Laboratory animals are species that are highly susceptible to alterations in thermoregulation due to their limited body surface area and the diverse stimuli and procedures to which they are subjected, which dictate the thermoregulation mechanisms that prevail. Therefore, it is essential to evaluate these alterations and the vasomotor responses that permit peripheral vasodilatation or vasoconstriction to facilitate thermal exchange. This is especially true for animals that are used in thermoregulation experimentation through vasomotor responses and heat production via BAT consumption under conditions of extreme cold.

According to the specific characteristics of different laboratory animals, the main body regions that are used are the orbital area, tail, auricular pavilion, and interscapular area or BAT. The temperature increases that are detected by IRT when applied to the ocular surface have been associated with stressful and painful events. In contrast, low temperatures in the tail are related to vasoconstriction in response to adverse stimuli, an effect that is similar to the one that is observed in the ears of rats and leporids. Finally, the interscapular window has shown greater usefulness for evaluating the thermogenesis capacity of small mammals under exposure to extreme cold.

Despite the evidence that is presented herein, the windows we addressed, and others that have been utilized with various species of laboratory animals, present differences that depend on species, experimental conditions, and even the characteristics of the instruments that are employed. The above reflects the simple fact that most of the animals that are used in laboratories belong to small species that move quickly, so the quality and definition of the devices employed must be sufficient to detect changes in the surface temperature of those subjects with great precision. This means that before validating a window to improve the interpretation of the information that is obtained by IRT, it is essential to assess these factors, the species, and the nature of the experiment to establish the degree of sensitivity and specificity for each body region that is examined to evaluate the health and thermostability of laboratory animals.

## Figures and Tables

**Table 1:** Different thermal windows and thermographic values in laboratory animals.

Reference	Research Objective	Species	Thermal Window	Basal Temperature (°C)	Experimental Phase Temperature (°C)	Ambiental Conditions
Fiebig et al. [57]	Compare rectal, subcutaneous, intraperitoneal temperature with infrared	Male NMRI nude mice (10 months old)	Dorsal region	37.36 ± 0.63	Compared to rectal temperature Mean difference of 0.56	22 °C ± 1 °C Relative humidity 53–56%
Vianna and Carrive [61]	Thermal response to conditioned fear	Male Wistar rats (350–500 g)	Regions of interest: Tail Paws Head Dorso Eyes	Basal 31.5 34.8 32.8 31.9 35.2	Fear 32.9 26.3 27.3 36.4 34.3	Ambient temperature 26–27 °C
Vogel et al. [53]	Thermal response to anesthesia with isoflurane	Mice C57BL/6J or CD-1	Ocular region	(1) 37.0 ± 0.3 (2) 37.3 ± 0.2 (3) 37.5 ± 0.2	35.6 ± 0.2 36.4 ± 0.2 36.9 ± 0.1	
Vogel et al. [53]	Comparison with rectal temperature and ocular surface	Mice C57BL/6J CD-1, Wistar rats and New Zealand White rab-bits	Ocular region	Rat T° Rectal 35.7 ± 0.1 Rabbit T° Rectal 38.2 ± 0.2 Mice T° rectal 37.5 ± 0.2	36.5 ± 0.2 39.1 ± 0.2 37.2 ± 0.2 °C	
Gjendal et al. [69]	Thermal response to three stressors (1) Anesthesia (2) Scruff (3) Peritoneal injection	Male C57BL/6 mice (n = 80)	Maximum temperature of Eye Tail Body Surface	Before isoflurane Eye: 37.32 Tail: 31.16 Body: 32.88 Scruff Eye: 37.04 Body: 32.4 Intraperitoneal Eye: 36.99 Body: 32.46	After isoflurane 34.70 28.13 30.29 37.25 32.13 37.19 32.25	Ambient temperature between 20–24 °C
Marks et al. [10]	Cold exposure	Wistar rats, weight 450– 550 g	Interscapular region Dorsal Tail	Average temperature Interscapular: 34.9 Dorsal: 34.4 Tail: 28.6	After exposure to cold (4 °C) 34.14 ± 0.17 30.96 ± 0.31 8.69 ± 0.49	Room temperature of 22–24 °C
Lecorps et al. [66]	Thermal response to predator odor 2,5-dihydro-2,4,5-trimethylthiazole (TMT), and water	Adult male house mice (4-month-old)	Tail Body surface	5 min before TMT 22.10 ± 0.9 °C 5 min before TMT 35.76 ± 0.08 °C	4 and 5 min after TMT, respectively 23.32 ± 1.3 °C 23.23 ± 1.65 °C 2, 3 and 4 min after TMT, respectively 35.90 ± 0.08 °C 35.98 ± 0.11 °C 36.03 ± 0.11 °C	A separated experimental room Reverse light-dark cycle
Franco et al. [98]	Hypothermia induced by lipopolysaccharides (*E. coli*)	Mice C57BL/6	Body surface Interscapular region	31.58 32.43		Ambient temperature at 20–24 °C
Całkosiński et al. [102]	Induced inflammatory response with carrageenan 1%	Female rats, 10-weeks-old, weight 150–160 g	Pleura Lower lip Right hindlimb Left hindlimb	Basal 34.00 ± 0.59 31.86 ± 0.57 24.66 ± 0.76 24.93 ± 0.85	72 h after the administration 35.91 ± 0.65 33.73 ± 0.66 26.26 ± 0.98 26.36 ± 0.82	Room temperature at 20 ± 1 °C
El Bitar et al. [33]	Thermoregulation assessment in an acute pain model Comparison between T_core_, T_ambient_ and T_tail_	Adult male Sprague-Dawley rats, 320–370 g	Tail	32.0 °C	Thermographic pictures every minute from a 20-min sequence 27.8	Experiments conducted between 9 am and 5 pm Ambient temperature 24.1 °C Mean core temperature of 38.2 °C
Nosrati et al. [49]	Measure disease activity in a collagen-induced rheumatoid arthritis model	Female 7-week-old DBA1/J mice 18–20 g	Wrist joints Front paws Ankle joints Hind paws Upper back	Quantified as temperature index TI_total_ (± SD) Baseline arthritis 3.37 ± 0.12 Baseline control 3.31 ± 0.06	Progressive increase at day 28 3.53–3.85 Same	Fluorescent light to prevent radiation Ambient temperature 24 ± 1°C
Brunell [60]	Compare IRT to rectal temperature	20 female and 30 male rhesus macaques, 3.5 to 11 years, weight of 3.4 to 11.7 kg	Chest Abdomen	Correlation coefficient with rectal temperature (first week) −0.079 −0.079	After approximately 3 weeks −0.012 0.060	Ambient temperature of 68 to 72 °F Relative humidity of 30 to 70% 12:12-h light:dark cycle
Farrar et al. [59]	Compare rectal an infrared thermometry	Female Yorkshire-cross swine, 12–15-week-old, 30–45 pounds	Area surrounding the eyes Neck Base of the ear	Mean baseline rectal 38 Eye basal ~37.1 °F Neck basal ~36.9 °F	~38.7 ~38.6 ~38.7	Distance between 24 and 32 inches

## Data Availability

Not applicable.

## References

[1] National Research Council (1988). Use of Laboratory Animals in Biomedical and Behavioral Research.

[2] Van Zutphen L.F.M.B. (2002). Use of animals in research: A science—Society controversy? The European perspective. ALTEX.

[3] Badyal D., Desai C. (2014). Animal use in pharmacology education and research: The changing scenario. Indian J. Pharmacol..

[4] Singh V.P., Pratap K., Sinha J., Desiraju K., Bahal D., Kukreti R. (2016). Critical evaluation of challenges and future use of animals in experimentation for biomedical research. Int. J. Immunopathol. Pharmacol..

[5] Casas-Alvarado A., Mota-Rojas D., Hernández-Ávalos I., Mora-Medina P., Olmos-Hernández A., Verduzco-Mendoza A., Reyes-Sotelo B., Martínez-Burnes J. (2020). Advances in infrared thermography: Surgical aspects, vascular changes, and pain monitoring in veterinary medicine. J. Thermal. Biol..

[6] Mota-Rojas D., Olmos-Hernández A., Verduzco-Mendoza A., Lecona-Butrón H., Martínez-Burnes J., Mora-Medina P., Gómez-Prado J., Orihuela A. (2021). Infrared thermal imaging associated with pain in laboratory animals. Exp. Anim..

[7] Rezende E.L., Bacigalupe L.D. (2015). Thermoregulation in endotherms: Physiological principles and ecological consequences. J. Comp. Physiol. B.

[8] Romanovsky A.A. (2014). Skin temperature: Its role in thermoregulation. Acta Physiol..

[9] Tan C.L., Knight Z.A. (2018). Regulation of body temperature by the nervous system. Neuron.

[10] Marks A., Vianna D.M.L., Carrive P. (2009). Nonshivering thermogenesis without interscapular brown adipose tissue involvement during conditioned fear in the rat. Am. J. Physiol. Integr. Comp. Physiol..

[11] Mota-Rojas D., Titto C.G., Orihuela A., Martínez-Burnes J., Gómez-Prado J., Torres-Bernal F., Flores-Padilla K., Carvajal-de la Fuente V., Wang D. (2021). Physiological and behavioral mechanisms of thermoregulation in mammals. Animals.

[12] Liedtke W.B. (2017). Deconstructing mammalian thermoregulation. Proc. Natl. Acad. Sci. USA.

[13] Škop V., Guo J., Liu N., Xiao C., Hall K.D., Gavrilova O., Reitman M.L. (2020). Mouse thermoregulation: Introducing the concept of the thermoneutral point. Cell Rep..

[14] Hankenson F.C., Marx J.O., Gordon C.J., David J.M. (2018). Effects of rodent thermoregulation on animal models in the research environment. Comp. Med..

[15] Gaskill B.N., Rohr S.A., Pajor E.A., Lucas J.R., Garner J.P. (2009). Some like it hot: Mouse temperature preferences in laboratory housing. Appl. Anim. Behav. Sci..

[16] Moberg G.P., Moberg G.P., Mench J.A. (2000). Biological response to stress: Implications for animal welfare. The Biology of Animal Stress: Basic Principles and Implications for Animal Welfare.

[17] Balcombe J.P., Barnard N.D., Sandusky C. (2004). Laboratory routines cause animal stress. Contemp. Top. Lab. Anim. Sci..

[18] Gaskill B.N., Garner J.P. (2017). Stressed out: Providing laboratory animals with behavioral control to reduce the physiological effects of stress. Lab Anim..

[19] Lim S., Honek J., Xue Y., Seki T., Cao Z., Andersson P., Yang X., Hosaka K., Cao Y. (2012). Cold-induced activation of brown adipose tissue and adipose angiogenesis in mice. Nat. Protoc..

[20] Nagashima K., Nakai S., Tanaka M., Kanosue K. (2000). Neuronal circuitries involved in thermoregulation. Auton. Neurosci..

[21] Morrison S.F., Nakamura K. (2019). Central mechanisms for thermoregulation. Annu. Rev. Physiol..

[22] Morrison S.F., Nakamura K., Madden C.J. (2008). Central control of thermogenesis in mammals. Exp. Physiol..

[23] Malheiros-Lima M.R., Pires W., Fonseca I.A.T., Joviano-Santos J.V., Ferreira A.J., Coimbra C.C., Lima N.R.V., Wanner S.P. (2018). Physical exercise-induced cardiovascular and thermoregulatory adjustments are impaired in rats subjected to cutaneous artery denervation. Front. Physiol..

[24] Angilletta M.J., Youngblood J.P., Neel L.K., VandenBrooks J.M. (2019). The neuroscience of adaptive thermoregulation. Neurosci. Lett..

[25] Da Scarpellini C.S., Cristina-Silva C., Biancardi V., Gargaglioni L.H., Almeida M.C., Bícego K.C. (2019). Hypothalamic TRPV4 channels participate in the medial preoptic activation of warmth-defence responses in Wistar male rats. Pflüg. Arch. Eur. J. Physiol..

[26] Yahiro T., Kataoka N., Nakamura Y., Nakamura K. (2017). The lateral parabrachial nucleus, but not the thalamus, mediates thermosensory pathways for behavioural thermoregulation. Sci. Rep..

[27] Abbott S.B.G., Saper C.B. (2018). Role of the median preoptic nucleus in the autonomic response to heat-exposure. Temperature.

[28] McAllen R.M., McKinley M.J. (2018). Efferent thermoregulatory pathways regulating cutaneous blood flow and sweating. Handbook of Clinical Neurology.

[29] Alba B.K., Castellani J.W., Charkoudian N. (2019). Cold-induced cutaneous vasoconstriction in humans: Function, dysfunction and the distinctly counterproductive. Exp. Physiol..

[30] Kamijo Y.-I., Lee K., Mack G.W. (2005). Active cutaneous vasodilation in resting humans during mild heat stress. J. Appl. Physiol..

[31] Fujii N., Kenny G.P., McGarr G.W., Amano T., Honda Y., Kondo N., Nishiyasu T. (2021). TRPV4 channel blockade does not modulate skin vasodilation and sweating during hyperthermia or cutaneous postocclusive reactive and thermal hyperemia. Am. J. Physiol. Integr. Comp. Physiol..

[32] Ootsuka Y., Blessing W.W. (2005). Inhibition of medullary raphé/parapyramidal neurons prevents cutaneous vasoconstriction elicited by alerting stimuli and by cold exposure in conscious rabbits. Brain Res..

[33] El Bitar N., Pollin B., Karroum E., Pincedé I., Mouraux A., Le Bars D. (2014). Thermoregulatory vasomotor tone of the rat tail and paws in thermoneutral conditions and its impact on a behavioral model of acute pain. J. Neurophysiol..

[34] Oladimeji A.M., Johnson T.G., Metwally K., Farghly M., Mahrose K.M. (2021). Environmental heat stress in rabbits: Implications and ameliorations. Int. J. Biometeorol..

[35] Johnson J.M., Kellogg D.L. (2018). Skin vasoconstriction as a heat conservation thermoeffector. Handbook of Clinical Neurology.

[36] Collier R.J., Gebremedhin K.G. (2015). Thermal biology of domestic animals. Annu. Rev. Anim. Biosci..

[37] Bovell D.L. (2018). The evolution of eccrine sweat gland research towards developing a model for human sweat gland function. Exp. Dermatol..

[38] Stevens L.M., Landis S.C. (1987). Development and properties of the secretory response in rat sweat glands: Relationship to the induction of cholinergic function in sweat gland innervation. Dev. Biol..

[39] Xu M., Wang D. (2015). Distribution and density of sweat glands in Mongolian gerbils (*Meriones unguiculatus*) and Brandt´s voles (*Lisiopodomys brandtii*). Acta Theriol. Sin..

[40] Labbé S.M., Caron A., Bakan I., Laplante M., Carpentier A.C., Lecomte R., Richard D. (2015). In vivo measurement of energy substrate contribution to cold-induced brown adipose tissue thermogenesis. FASEB J..

[41] Labbé S.M., Caron A., Lanfray D., Monge-Rofarello B., Bartness T.J., Richard D. (2015). Hypothalamic control of brown adipose tissue thermogenesis. Front. Syst. Neurosci..

[42] Tupone D., Madden C.J., Morrison S.F. (2014). Autonomic regulation of brown adipose tissue thermogenesis in health and disease: Potential clinical applications for altering BAT thermogenesis. Front. Neurosci..

[43] Vialard F., Olivier M. (2020). Thermoneutrality and Immunity: How does cold stress affect disease?. Front. Immunol..

[44] Gordon C.J. (2012). Thermal physiology of laboratory mice: Defining thermoneutrality. J. Therm. Biol..

[45] Terrien J. (2011). Behavioral thermoregulation in mammals: A review. Front. Biosci..

[46] Gaskill B.N., Gordon C.J., Pajor E.A., Lucas J.R., Davis J.K., Garner J.P. (2013). Impact of nesting material on mouse body temperature and physiology. Physiol. Behav..

[47] Gaskill B.N., Gordon C.J., Pajor E.A., Lucas J.R., Davis J.K., Garner J.P. (2012). Heat or insulation: Behavioral titration of mouse preference for warmth or access to a nest. PLoS ONE.

[48] Nääs I.A., Garcia R.G., Caldara F.R. (2014). Infrared thermal image for assessing animal health and welfare. J. Anim. Behav. Biometeorol..

[49] Nosrati Z., Bergamo M., Rodríguez-Rodríguez C., Saatchi K., Häfeli U.O. (2020). Refinement and validation of infrared thermal imaging (IRT): A non-invasive technique to measure disease activity in a mouse model of rheumatoid arthritis. Arthritis Res. Ther..

[50] Van der Vinne V., Pothecary C.A., Wilcox S.L., McKillop L.E., Benson L.A., Kolpakova J., Tam S.K.E., Krone L.B., Fisk A.S., Wilson T.S. (2020). Continuous and non-invasive thermography of mouse skin accurately describes core body temperature patterns, but not absolute core temperature. Sci. Rep..

[51] Mota-Rojas D., Pereira M.F.A., Wang D., Martínez-Burnes J., Ghezzi M., Hernández-Ávalos I., Lendez P., Mora-Medina P., Casas A., Olmos-Hernández A. (2021). Clinical applications and factors involved in validating thermal windows in large rumiants to assess health and productivity. Animals.

[52] Tattersall G.J. (2016). Infrared thermography: A non-invasive window into thermal physiology. Comp. Biochem. Physiol. Part A Mol. Integr. Physiol..

[53] Vogel B., Wagner H., Gmoser J., Wörner A., Löschberger A., Peters L., Frey A., Hofmann U., Frantz S. (2016). Touch-free measurement of body temperature using close-up thermography of the ocular surface. MethodsX.

[54] International Committee on Veterinary Gross Anatomical Nomenclature (2017). Nomina Anatomica Veterinaria.

[55] Gordon C.J., Puckett E., Padnos B. (2002). Rat tail skin temperature monitored noninvasively by radiotelemetry: Characterization by examination of vasomotor responses to thermomodulatory agents. J. Pharmacol. Toxicol. Methods.

[56] Gordon C.J. (1990). Thermal biology of the laboratory rat. Physiol. Behav..

[57] Fiebig K., Jourdan T., Kock M.H., Merle R., Thöne-Reineke C. (2018). Evaluation of infrared thermography for temperature measurement in adult male NMRI nude mice. J. Am. Assoc. Lab. Anim. Sci..

[58] Conley K.E., Porter W.P. (1985). Heat loss regulation: Role of appendages and torso in the deer mouse and the white rabbit. J. Comp. Physiol. B.

[59] Farrar K.L., Field A.E., Norris S.L., Jacobsen K.O. (2020). Comparison of rectal and infrared thermometry temperatures in anesthetized swine (*Sus scrofa*). J. Am. Assoc. Lab. Anim. Sci..

[60] Brunell M.K. (2012). Comparison of noncontact infrared thermometry and 3 commercial subcutaneous temperature transponding microchips with rectal thermometry in rhesus macaques (*Macaca mulatta*). J. Am. Assoc. Lab. Anim. Sci..

[61] Vianna D.M.L., Carrive P. (2005). Changes in cutaneous and body temperature during and after conditioned fear to context in the rat. Eur. J. Neurosci..

[62] Seidel J., Bockhop F., Mitkovski M., Martin S., Ronnenberg A., Krueger-Burg D., Schneider K., Röhse H., Wüstefeld L., Cosi F. (2020). Vascular response to social cognitive performance measured by infrared thermography: A translational study from mouse to man. FASEB BioAdv..

[63] Pereira C., Kunczik J., Zieglowski L., Tolba R., Abdelrahman A., Zechner D., Vollmar B., Janssen H., Thum T., Czaplik M. (2018). Remote welfare monitoring of rodents using thermal imaging. Sensors.

[64] Weitkamp J. (2020). Effect of Tickling and Gentling on Eye and Tail Temperature of Laboratory Rats during Manual Restraint, Using Infrared Thermography. Master’s Thesis.

[65] Luong L.N.L., Carrive P. (2012). Orexin microinjection in the medullary raphe increases heart rate and arterial pressure but does not reduce tail skin blood flow in the awake rat. Neuroscience.

[66] Lecorps B., Rödel H.G., Féron C. (2019). Short-term thermal responses after exposure to predator odor (TMT) in the house mouse. Mamm. Biol..

[67] Mota-Rojas D., Wang D., Titto C.G., Gómez-Prado J., Carvajal-de la Fuente V., Ghezzi M., Boscato-Funes L., Barrios-García H., Torres-Bernal F., Casas-Alvarado A. (2021). Pathophysiology of Fever and Application of Infrared Thermography (IRT) in the Detection of Sick Domestic Animals: Recent Advances. Animals.

[68] Bell C., Rogers S., Taylor J., Busby D. (2019). Improving the recognition of equine affective states. Animals.

[69] Gjendal K., Franco N.H., Ottesen J.L., Sørensen D.B., Olsson I.A.S. (2018). Eye, body or tail? Thermography as a measure of stress in mice. Physiol. Behav..

[70] Wirth S., Gebhardt-Henrich S., Riemer S., Hattendorf J., Zinsstag J., Hediger K. (2020). The influence of human interaction on guinea pigs: Behavioral and thermographic changes during animal-assisted therapy. Physiol. Behav..

[71] Stewart M., Stookey J.M., Stafford K.J., Tucker C.B., Rogers A.R., Dowling S.K., Verkerk G.A., Schaefer A.L., Webster J.R. (2009). Effects of local anesthetic and a nonsteroidal antiinflammatory drug on pain responses of dairy calves to hot-iron dehorning. J. Dairy Sci..

[72] Nijland N. (2021). The Influence of Different Types of Behaviour on the Eye Temperature of Mice Using Infrared Thermograohy. Master’s Thesis.

[73] Ding J.E., Kim Y.H., Yi S.M., Graham A.D., Li W., Lin M.C. (2021). Ocular surface cooling rate associated with tear film characteristics and the maximum interblink period. Sci. Rep..

[74] Lecorps B., Rödel H.G., Féron C. (2016). Assessment of anxiety in open field and elevated plus maze using infrared thermography. Physiol. Behav..

[75] Maynard R.L., Downes N., Maynard R., Downes N. (2019). Cardiovascular System. Anatomy and Histology of the Labor.

[76] Moore D.M., Zimmerman K., Smith S.A. (2015). Hematological assessment in pet rabbits. Vet. Clin. N. Am. Exot. Anim. Pract..

[77] Ludwig N., Gargano M., Luzi F., Carenzi C., Verga M. (2007). Technical note: Applicability of infrared thermography as a non invasive measurements of stress in rabbit. World Rabbit Sci..

[78] McCafferty D.J. (2007). The value of infrared thermography for research on mammals: Previous applications and future directions. Mamm. Rev..

[79] Hutu I., Patras I., Gherghel D., Lungu B. (2018). Application of infrared thermograohy in rabbit orthopaedic models. Proceedings of the Life Sciences, a Challenge for the Future.

[80] Redaelli V., Luzi F., Verga M. Infrared hermography (IRT) in nude mice: An alternative method for body temperature measurement. Proceedings of the Annual Meeting.

[81] De Lima V., Piles M., Rafel O., López-Béjar M., Ramón J., Velarde A., Dalmau A. (2013). Use of infrared thermography to assess the influence of high environmental temperature on rabbits. Res. Vet. Sci..

[82] Luzi F., Ludwig N., Gargano M., Milazzo M., Carenzi C., Verga M. (2007). Evaluation of skin temperature change as stress indicator in rabbit through infrared thermography. Ital. J. Anim. Sci..

[83] Gilbert C., McCafferty D.J., Giroud S., Ancel A., Blanc S. (2012). Private heat for public warmth: How huddling shapes individual thermogenic responses of rabbit pups. PLoS ONE.

[84] Wokke E.S. (2017). Refinement: Evaluating Stress and Accuracy of Different Intraperitoneal Techniques in Mice. Master’s Thesis.

[85] Xu Z., Agbigbe O., Nigro N., Yakobi G., Shapiro J., Ginosar Y. (2021). Use of high-resolution thermography as a validation measure to confirm epidural anesthesia in mice: A cross-over study. Int. J. Obstet. Anesth..

[86] Schaefer A.L., Cook N., Tessaro S.V., Deregt D., Desroches G., Dubeski P.L., Tong A.K.W., Godson D.L. (2004). Early detection and prediction of infection using infrared thermography. Can. J. Anim. Sci..

[87] Pastorelli G., Faustini M., Luzi F., Redaelli V., Turin L. (2020). *Passiflora Incarnata* powder extract in postweaning piglets feeding slightly improves wellbeing and immune parameters. Livest. Sci..

[88] Mrzilkova J., Michenka P., Seremeta M., Kremen J., Dudak J., Zemlicka J., Musil V., Minnich B., Zach P. (2020). Morphology of the vasculature and blood supply of the brown adipose tissue examined in an animal model by micro-CT. Biomed Res. Int..

[89] Warner A., Kjellstedt A., Carreras A., Böttcher G., Peng X.-R., Seale P., Oakes N., Lindén D. (2016). Activation of β 3 -adrenoceptors increases in vivo free fatty acid uptake and utilization in brown but not white fat depots in high-fat-fed rats. Am. J. Physiol. Metab..

[90] Farrell W.J., Alberts J.R. (2000). Ultrasonic vocalizations by rat pups after adrenergic manipulations of brown fat metabolism. Behav. Neurosci..

[91] Boileau A., Farish M., Turner S.P., Camerlink I. (2019). Infrared thermography of agonistic behaviour in pigs. Physiol. Behav..

[92] Redaelli V., Papa S., Marsella G., Grignaschi G., Bosi A., Ludwig N., Luzi F., Vismara I., Rimondo S., Veglianese P. (2019). A refinement approach in a mouse model of rehabilitation research. Analgesia strategy, reduction approach and infrared thermography in spinal cord injury. PLoS ONE.

[93] Jaén-Téllez J.A., Sánchez-Guerrero M.J., López-Campos J.I., Valera M., González-Redondo P. (2020). Acute stress assessment using infrared thermography in fattening rabbits reacting to handling under winter and summer conditions. Span. J. Agric. Res..

[94] Brzezinski R.Y., Ovadia-Blechman Z., Lewis N., Rabin N., Zimmer Y., Levin-Kotler L., Tepper-Shaihov O., Naftali-Shani N., Tsoref O., Grossman E. (2019). Non-invasive thermal imaging of cardiac remodeling in mice. Biomed. Opt. Express.

[95] Mei J., Riedel N., Grittner U., Endres M., Banneke S., Emmrich J.V. (2018). Body temperature measurement in mice during acute illness: Implantable temperature transponder versus surface infrared thermometry. Sci. Rep..

[96] Song C., Appleyard V., Murray K., Frank T., Sibbett W., Cuschieri A., Thompson A. (2007). Thermographic assessment of tumor growth in mouse xenografts. Int. J. Cancer.

[97] Vainer B.G. (2018). A Novel High-Resolution Method for the Respiration Rate and Breathing Waveforms Remote Monitoring. Ann. Biomed. Eng..

[98] Franco N.H., Gerós A., Oliveira L., Olsson I.A.S., Aguiar P. (2019). ThermoLabAnimal—A high-throughput analysis software for non-invasive thermal assessment of laboratory mice. Physiol. Behav..

[99] Mota-Rojas D., Titto C.G., Geraldo A.M., Martínez-Burnes J., Gómez J., Hernández-Ávalos I., Casas A., Domínguez A., José N., Bertoni A. (2021). Efficacy and function of feathers, hair, and glabrous skin in the thermoregulation strategies of domestic animals. Animals.

[100] Mazur-Milecka M., Kocejko T., Ruminski J. (2020). Deep instance segmentation of laboratory animals in thermal images. Appl. Sci..

[101] Unger S.D., Rollins M.A., Thompson C.M. (2020). Hot- or Cold-Blooded? A Laboratory Activity That Uses Accessible Technology to Investigate Thermoregulation in Animals. Am. Biol. Teach..

[102] Całkosiński I., Dobrzyński M., Rosińczuk J., Dudek K., Chrószcz A., Fita K., Dymarek R. (2015). The use of infrared thermography as a rapid, quantitative, and noninvasive method for evaluation of inflammation response in different anatomical regions of rats. Biomed. Res. Int..

